# Mitochondrial stress delays tumorigenesis in a Li-Fraumeni syndrome mouse model

**DOI:** 10.15698/cst2019.04.182

**Published:** 2019-03-21

**Authors:** Matthew P. Donnelly, Jie Li, Paul M. Hwang

**Affiliations:** 1 Cardiovascular Branch, National Heart, Lung and Blood Institute, National Institutes of Health, Bethesda, Maryland, USA.

**Keywords:** p53, mitochondria, Li-Fraumeni syndrome, mouse model, cyclin D1, DNA polymerase gamma, metformin

As a prominent hub of metabolic activity, the mitochondrion can initiate retrograde signaling in response to bioenergetic stress that is important for various adaptive changes including cell cycle regulation [1, 2]. There is abundant evidence that cancer cells display alterations in metabolism, such as increased mitochondrial fatty acid oxidation [3, 4], while noncanonical functions of p53 including regulation of redox metabolism appear sufficient for its tumor suppressive activity [5]. Therefore, we hypothesized that targeted regulation of mitochondrial respiration may have beneficial anti-tumorigenic effects through induction of cellular programs that limit cell growth promoted by mutant p53. To test this, we performed both genetic and pharmacological disruption of the mitochondria in a mouse model of Li-Fraumeni Syndrome (LFS), a cancer predisposition disorder characterized by germline mutations in *TP53* that can enhance mitochondrial metabolism [6, 7]. Overall, the partial inhibition of mitochondrial respiration both through mutation of DNA polymerase γ (Polg) and treatment with the well-known antidiabetic drug metformin improved cancer-free survival in LFS mice through a conserved mechanism that induced autophagy and an anti-proliferative cell signaling program.

We have previously reported that an LFS mouse model with the p53 R172H knockin mutation (*p53*^*R172H*^), homologous to the human p53 R175H point mutation, can increase oxidative metabolism and promote tumorigenesis through increased cellular proliferation [6]. To examine whether genetic modification of mitochondrial respiration can affect cancer progression, we crossed LFS mice with mice bearing the D257A mutation in DNA polymerase γ (*Polg*^*mut*^), which are prone to frequent mitochondrial DNA mutations and develop mitochondrial dysfunction. Accordingly, introducing the *Polg*^*mut*^ into the *p53*^*R172H*^ background caused mutant allele dose-dependent decreases in oxygen consumption rate in thymic tissue (where tumors arise) and concomitant increases in markers of glycolysis such as blood lactate, likely in compensation for decreased oxidative phosphorylation. Remarkably, this metabolic shift was associated with increased cancer-free survival. The observation that survival time of individual mice positively correlated with blood lactate level suggested that, within a physiologically adaptable range, the degree of mitochondrial inhibition dictates its tumor preventive effect in a germline p53 mutation setting.

We next sought to identify cell signaling changes in response to mitochondrial inhibition associated with cancer prevention to serve as biomarkers. Because mutant p53 can inhibit autophagy, we examined the level of lipidated autophagosome membrane associated protein LC3-II. As expected, *p53*^*R172H*^ mice exhibited decreased levels of LC3-II in thymus tissue compared with wild-type mice, which was rescued with the introduction of mutant Polg. Autophagy in concert with endoplasmic reticulum stress have been shown to result in the activation of glycogen synthase kinase 3α/β (GSK3) through tyrosine autophosphorylation, and activated GSK3 can downregulate the cell cycle promoter cyclin D1 [8]. Given the induction of LC3-II, we examined whether these anti-proliferative cell signaling mediators were affected by the inhibition of mitochondrial respiration. *p53*^*R172H*^ mice had significantly lower activated GSK3 as indicated by decreased tyrosine autophosphorylation and increased cyclin D1 levels. However, when the Polg mutant allele was introduced into the *p53*^*R172H*^ genetic background, the double mutant state displayed increased GSK3 activation and decreased cyclin D1 expression despite the presence of mutant p53.

To translate these findings made from the genetic inhibition of mitochondria, we tested the anti-diabetic drug metformin for potential therapeutic application in LFS given its previously described property of inhibiting respiration as well as extensive history of safety and benefit in humans. Metformin recapitulated autophagy induction, GSK3 activation, cyclin D1 repression, and cell growth inhibition in human colon cancer HCT116 cells in a dose-dependent manner [7]. We next treated *p53*^*R172H*^ mice with intraperitoneal doses of metformin that have been reported to result in plasma concentrations equivalent to human therapeutic levels. The treatment with metformin reproduced the anti-proliferative cell signaling observed in tissue culture, establishing the signaling pathway as a biomarker of mitochondrial regulated cell signaling.

Administering metformin in the drinking water of LFS mice at human-equivalent therapeutic doses after weaning was also sufficient to decrease mitochondrial respiration and activate the anti-proliferation signaling cascade in thymus tissue. This treatment resulted in a significant 27% increase in mean cancer-free survival time. To examine the effect of metformin treatment in patients with LFS, we carried out a pilot study that examined mitochondrial function and the presence of the previously established anti-proliferative biomarkers before, during, and after treatment. Metformin administered at the recommended treatment dose of 2000 mg daily disrupted mitochondrial respiration as evidenced through decreased oxygen consumption in freshly isolated blood mononuclear cells after 8-14 weeks of treatment. Previously, we established that the noninvasive measurement of skeletal muscle phosphocreatine (PCr) recovery kinetics after exercise using P-31 magnetic resonance spectroscopy is an indicator of *in vivo* oxidative phosphorylation capacity in LFS [6]. As mitochondrial activity is necessary for the regeneration of PCr after exercise, the increase in the PCr recovery time constant seen in LFS patients at week 8 and 14 following metformin administration provided further evidence of decreased mitochondrial respiration *in vivo*. Remarkably, this *in vivo* perturbation of mitochondrial respiration with metformin showed the conserved signaling program of increased autophagy, activated GSK3, and downregulated cyclin D1 in humans.

**Figure 1:**
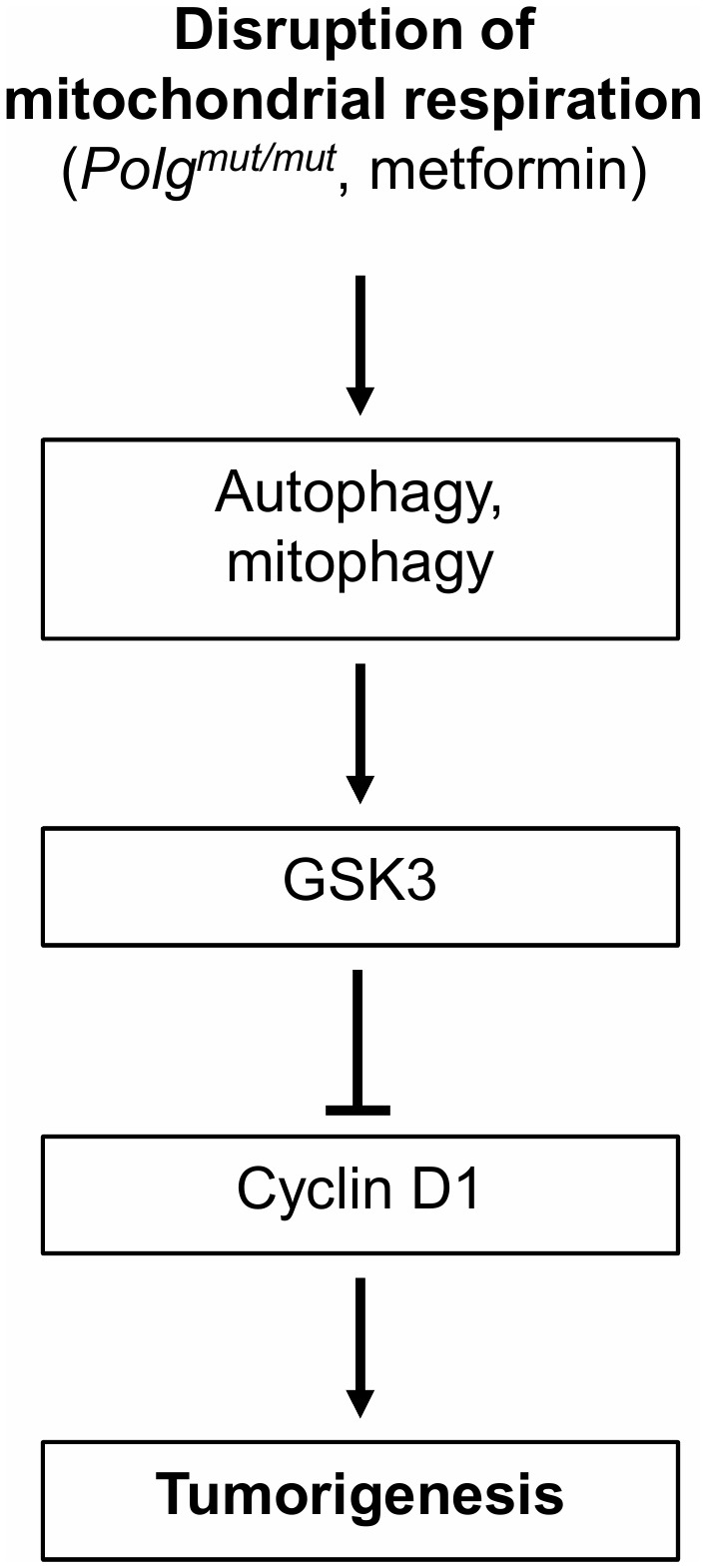
FIGURE 1: Proposed anti-proliferative signaling mechanism in response to mitochondrial inhibition. Inhibition of mitochondrial respiration genetically (*Polg*^*mut/mut*^) or pharmacologically (metformin) can delay tumorigenesis in association with induction of autophagy and GSK3-mediated suppression of cyclin D1 to affect cell cycle progression.

This study demonstrates that inhibition of mitochondrial respiration, either genetically or pharmacologically, is capable of preventing tumorigenesis and increasing cancer-free survival time in association with the induction of autophagy and anti-proliferative cell stress signaling (**Figure 1**). It suggests that mitochondrial function is intricately tied with cancer progression. Of particular note is the positive correlation between plasma lactate level, indicative of the degree of mitochondrial inhibition, and cancer-free survival time in the p53 mutant mice. It provides further support for the increasing evidence that mitochondrial function is important for cancer progression, albeit contrary to the Warburg theory of cancer [9]. Although mitochondrial inhibition does induce cellular stress and metabolic reprogramming, it appears to be beneficial in the cancer prone genetic background of LFS.

Metformin is known to have pleiotropic cellular effects, so tumor suppression upon treatment is likely to occur through multiple mechanisms ranging from senescence to immune regulation [10]. Co-inhibition of CDK4/6 and MEK has recently been shown to promote the clearance of tumor cells by enhancing natural killer (NK) cell function [11]. As MEK is known to upregulate cyclin D1, perhaps repression of cyclin D1 by the metformin-induced anti-proliferative cell signaling pathway contributes to NK-mediated cell clearance. A recent *in vitro* report has also supported our finding by showing that metformin prevents cell tumorigenesis in association with ER stress and autophagy-induced cell death [12]. It could be speculated that the delay in cancer progression by mitochondrial stress signaling may extend beyond the mutant p53 state, but based on these preliminary findings, more studies are necessary to determine the cancer preventive efficacy of metformin in other conditions.

Other than avoiding ionizing radiation, there are currently no cancer preventive measures in Li-Fraumeni syndrome indicating an urgent need for further clinical investigations. From a translational perspective, administering metformin to mice at 4 weeks of age is equivalent to starting treatment in a 14 year old human. Although the use of metformin may not obviate the risk of early pediatric cancers associated with LFS, subsequent cancers, especially early onset breast cancer in women, as well as later onset cancers could be considered for chemoprevention. A randomized placebo-controlled study of metformin for cancer prevention in LFS may be difficult to conduct, but it is imperative to consider performing modified chemoprevention trials in LFS to affect cancer outcome and improve survival, whether this involves metformin or another promising therapy.
